# Continuous Release of Tumor-Derived Factors Improves the Modeling of Cachexia in Muscle Cell Culture

**DOI:** 10.3389/fphys.2017.00738

**Published:** 2017-09-25

**Authors:** Robert W. Jackman, Jess Floro, Rei Yoshimine, Brian Zitin, Maythita Eiampikul, Khalid El-Jack, Danielle N. Seto, Susan C. Kandarian

**Affiliations:** Department of Health Sciences, Boston University Boston, MA, United States

**Keywords:** muscle atrophy, myotubes, LIF, muscle wasting, C2C12, cancer, cachexia, transwell

## Abstract

Cachexia is strongly associated with a poor prognosis in cancer patients but the biological trigger is unknown and therefore no therapeutics exist. The loss of skeletal muscle is the most deleterious aspect of cachexia and it appears to depend on secretions from tumor cells. Models for studying wasting in cell culture consist of experiments where skeletal muscle cells are incubated with medium conditioned by tumor cells. This has led to candidates for cachectic factors but some of the features of cachexia *in vivo* are not yet well-modeled in cell culture experiments. Mouse myotube atrophy measured by myotube diameter in response to medium conditioned by mouse colon carcinoma cells (C26) is consistently less than what is seen in muscles of mice bearing C26 tumors with moderate to severe cachexia. One possible reason for this discrepancy is that *in vivo* the C26 tumor and skeletal muscle share a circulatory system exposing the muscle to tumor factors in a constant and increasing way. We have applied Transwell®-adapted cell culture conditions to more closely simulate conditions found *in vivo* where muscle is exposed to the ongoing kinetics of constant tumor secretion of active factors. C26 cells were incubated on a microporous membrane (a Transwell® insert) that constitutes the upper compartment of wells containing plated myotubes. In this model, myotubes are exposed to a constant supply of cancer cell secretions in the medium but without direct contact with the cancer cells, analogous to a shared circulation of muscle and cancer cells in tumor-bearing animals. The results for myotube diameter support the idea that the use of Transwell® inserts serves as a more physiological model of the muscle wasting associated with cancer cachexia than the bolus addition of cancer cell conditioned medium. The Transwell® model supports the notion that the dose and kinetics of cachectic factor delivery to muscle play a significant role in the extent of pathology.

## Background

Cachexia is a devastating consequence of cancer in ~50% of patients (Tisdale, 2009). It is characterized by elevated metabolism and whole body wasting that is not reversed by feeding (Fielitz, 2016). The loss of skeletal muscle causes the most deleterious health consequences such as weakness, fatigue, metabolic instability, and therefore, an inability to tolerate medical treatment (Tisdale, 2009). Muscle loss in cancer cachexia has received more attention recently as it becomes clear that it is the best indicator of a poor prognosis (Martin et al., 2013). However, our understanding of the mechanisms underlying cancer cachexia is in its infancy, and currently no treatment exists.

One consensus of the etiology of cachexia is that it is initiated and/or maintained by secretions from the cells of the tumor (Coletti et al., 2006; Fearon et al., 2012; Fielitz, 2016). Therefore, many investigations have been carried out to determine the factor/s from different tumor cells that contribute to wasting. Among the many models for these studies are cell culture experiments in which cachexia target cells, such as skeletal muscle, are incubated with medium conditioned by tumor cells (Penna et al., 2016). Therefore, cancer cell conditioned medium (CM) treatment of target cells has been used as an *in vitro* approach to study the mechanism of wasting in muscle cells (Zhang et al., 2011; Puppa et al., 2014; Silva et al., 2015; Bohnert et al., 2016; Fukawa et al., 2016).

Results from studies using conditioned medium to understand the mechanism of myotube atrophy have shown involvement of a number of cancer cell secreted factors (TNFα, IL-6, LIF), and target cell signaling proteins and transcription factors such as C/EBPβ, C/EBPδ, STAT3, P38 (Zhang et al., 2011; Puppa et al., 2014; Seto et al., 2015; Silva et al., 2015; Bohnert et al., 2016; Fukawa et al., 2016). Differences in the molecules found to be involved in the mechanism of wasting are likely due to the tumor type (Penna et al., 2016). In an in-depth study, use of conditioned medium made from the commonly used C26 mouse tumor cell line led to the identification of LIF as the secreted peptide required for myotube atrophy (Seto et al., 2015). We showed that LIF was acting through the JAK2/STAT3 pathway to effect atrophy. These data were consistent with what we found in mice harboring C26 tumors. In cell culture, LIF from the C26 cells was entirely responsible for the atrophy effect of cancer cell conditioned medium on C2C12 myotubes.

Although, secretions from tumor cells have been useful to identify factors involved in muscle wasting, some of the features of cachexia *in vivo* are not yet well-modeled in cell culture (Penna et al., 2016). For instance, mouse myotube atrophy measured by myotube diameter in response to C26 CM is consistently less than what is seen by muscle cross-sectional area in mice with moderate to severe cachexia where the C26 tumor and muscle share a circulatory system (Bonetto et al., 2011; Cornwell et al., 2014; Seto et al., 2015). In addition, serum levels of LIF in cachectic C26 tumor-bearing mice were relatively less than that used in the cell culture model, yet muscle atrophy was greater (Seto et al., 2015). We wondered whether the modification of cell culture conditions to more closely simulate conditions found *in vivo* where muscle is exposed to ongoing kinetics of constant tumor secretion of active factors might improve cell culture models. Therefore, the purpose of this study is to determine the extent and progression of myotube atrophy due to 3 days exposure to secretions from C26 tumor cells delivered as a bolus as commonly used in conditioned medium studies, compared to myotubes plated in a well containing cancer cells in an upper compartment of the well. That is, C26 cells were incubated on a microporous membrane (a Transwell® insert, see Renaud and Martinoli, 2016) that constitutes the upper compartment of wells containing plated myotubes. In this latter model, myotubes are exposed to a constant supply of cancer cell secretions in the medium but without direct contact with the cancer cells, analogous to a shared circulation of muscle and cancer cells in tumor-bearing animals. Therefore, the use of a Transwell® plate, although perhaps not recreating the volume of shared fluid, should recapitulate the delay for tumor cell secretion and binding of cachectic factors to myotubes. In fact, the results support the idea that the use of Transwell® inserts serves as a more physiological model of the muscle wasting associated with cancer cachexia than the use of cancer cell conditioned medium as is customarily used.

## Materials and methods

### Cells

C2C12 cells were from ATCC and used within the first 15 passages. C26 mouse adenocarcinoma cells were obtained from the National Cancer Institute (Frederick, MD). Ehrlich tumor cells were obtained from ATCC. All cell culture reagents were from Invitrogen, Carlsbad, CA, USA.

### Media and cell culture

Cancer cells were plated and maintained at 37C, 5% CO2 in Dulbecco's Modified Eagle Medium, high glucose (supplemented with 10% fetal bovine serum, 1% penicillin-streptomycin, and 1% L-glutamine). C2C12 cells were serially cultured in the same medium. Differentiation medium (DM) was used for making conditioned medium from the tumor cells and for differentiating the C2C12 cells. DM consisted of DMEM with 2% horse serum with antibiotics and glutamine.

### Conditioned medium (CM) collection

Tumor cells were grown in culture medium as described. Once the plates reached a confluency of >90%, the growth medium was removed, and the cells were washed twice with sterile PBS and incubated in DM. CM was collected in DM on the tumor cells because the Transwell® plates were going to be cultured in DM to maintain differentiation of the C2C12 myotubes in the wells. After 24 h, the medium was collected and centrifuged in 50 ml falcon tubes at 4,500 rpm for 15 min in 4°C. Aliquots of the cell-cleared medium were stored at −80 C for up to a year.

### Treatment of myotubes with CM

C2C12 myoblasts in growth serum were plated on a 6-well plate at a density of 5 × 10^5^ cells/well and left for overnight attachment. The medium was then switched to differentiation medium, and the cells were differentiated for 4 days. Conditioned medium treatments were begun at a dilution of 33% CM in DM. Controls were with DM alone. At the beginning and end of each of three 24 h periods, medium was sampled for LIF measurement and photographs were taken for determining myotube diameters. At each 24 h interval after that, the plates were again photographed and medium sampled and the medium was replaced with fresh control or tumor cell-conditioned media.

### Transwell® insert protocol

The Transwell® system for all experiments consisted of Costar #3450 6-well plates with 0.4 micron porous inserts made of tissue culture treated polystyrene (https://www.corning.com/worldwide/en/products/life-sciences/products/permeable-supports/transwell-snapwell-netwell-falcon-permeable-supports.html). C2C12 cells were plated onto the bottom of the wells in the 6-well plate in the same way as for the CM treatments and then differentiated for 4 days. Tumor cells were plated onto the inserts in separate 6-well plates from those with the C2C12 cells. The tumor cells were approximately confluent at the time that the C2C12 myotubes were differentiated. The medium for the tumor cells on the Transwells was changed to DM for the last 2 days of culture before adding the insert wells to the myotube 6-well plates. Therefore, on day 4 of differentiation the myotube wells were photographed as for the CM treatment experiments and all medium was replaced with DM and the insert wells were placed above differentiated myotube wells. This was called day 0. Medium was sampled from the lower chamber at 0 h and at 1, 2, 4, 8, 12, and 24 h later. The sampling for Transwells was more frequent than for the CM experiments in order to be sure of the kinetics of LIF movement from the upper to lower chambers. The medium was changed at 24 and 48 h and the same sampling was done for each 24 h period and also photographs of the myotube wells were made daily for 3 days. Note: Tumor cells are normally grown in DMEM with 10% FCS, but they also grow well in less serum (i.e., DM).

### Myotube diameter measurement

At each time of myotube photography, 20X magnified phase micrographs were taken. Using a Nikon TS-5000 inverted microscope coupled with a Spot RT camera and Spot Software (Diagnostic Instruments), seven photos of each well were taken. The diameter of each myotube was an average of 3 measurements along the cell and at least 150 myotubes were measured per group (time point and treatment) using MetaMorph Imaging software (Universal Imaging). These data were coupled to excel spreadsheets and graphed using Prism software.

### ELISA

The ELISA for mouse LIF was from R&D (Minneapolis, MN, USA) and was performed according to the manufacturer's protocol.

### Statistics

For one variable analysis, an unpaired *t*-test was used to determine statistical difference at each time point. Data are expressed as means ± SEM, and statistical difference was defined at *P* < 0.05 as noted in the figure legends.

## Results

### LIF levels in the medium of myotube cultures treated with bolus delivery of C26 CM or with C26 cells plated in a transwell® insert

**Figure 1:**
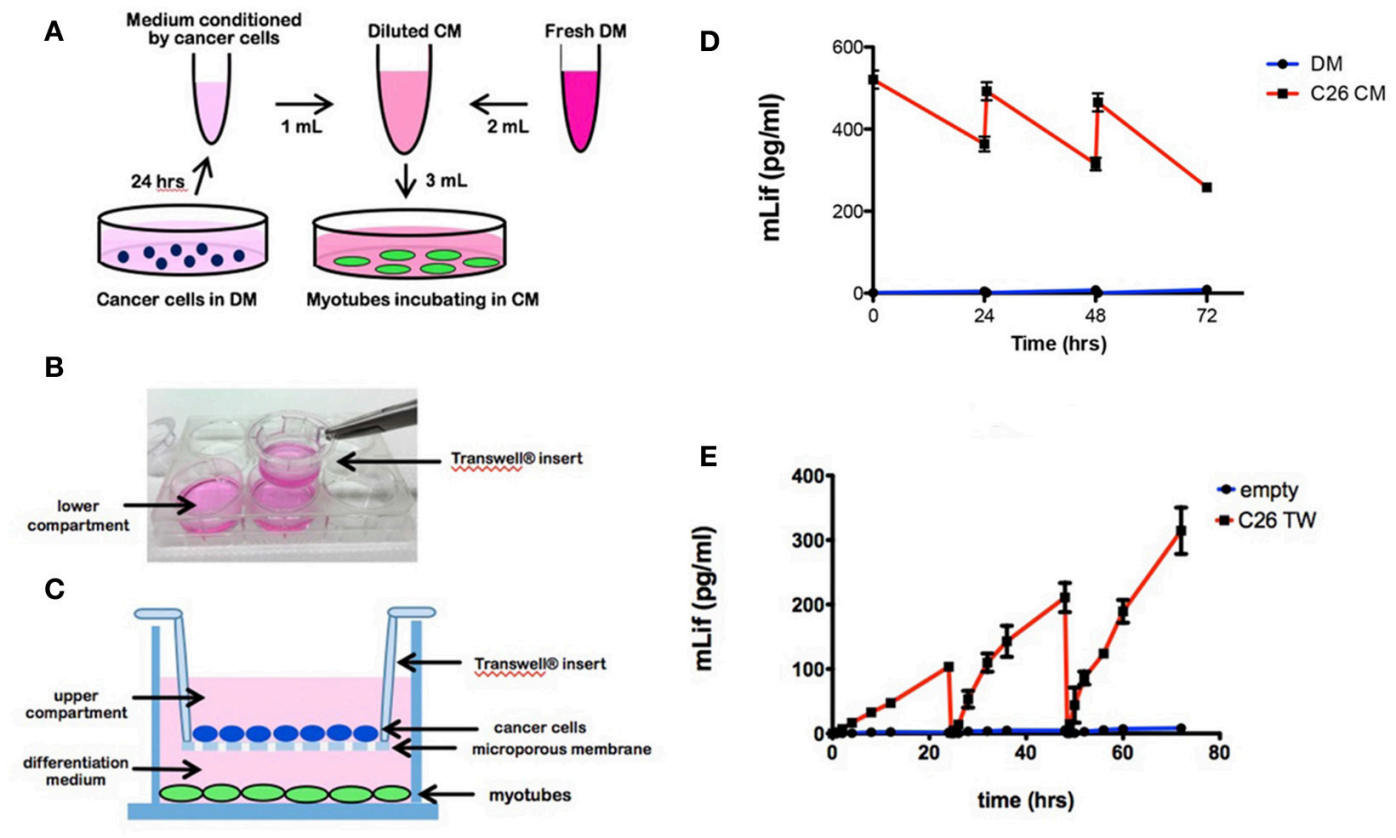
Cell culture models of tumor cell treated myotubes and the associated LIF levels in medium. **(A)** Schematic drawing of the design for the conventional conditioned medium (CM) model. C26 cancer cells were incubated in differentiation medium (DM) for 24 h to produce conditioned medium. Four day-differentiated C2C12 myotubes were treated for 72 h with 33% CM in fresh DM, refreshed every 24 h. **(B)** Photograph of a Transwell insert being placed into a well of a 6 well plate. **(C)** Schematic diagram of the Transwell system with C26 cancer cells in the upper compartment and C2C12 4-day myotubes growing below (figure is redrawn from Corning website). Cancer cells were seeded on microporous membrane insert (0.4 μm) placed into the well containing myotubes for 72 h with the upper and lower compartments containing DM, refreshed every 24 h. **(D)** Graph of LIF levels in medium from myotube cultures treated with DM (control) or C26 CM at the beginning and end of each 24 h period of treatment. **(E)** Graph of LIF levels in the lower compartment of myotube cultures with Transwell (TW) inserts seeded with C26 cells. “Empty” indicates control myotube cultures containing Transwell inserts without C26 cells.

The conventional experimental approach for treatment of 4-day myotubes with medium conditioned by cancer cells added as a bolus and diluted in differentiation medium (DM) is shown in a schematic diagram (Figure 1A). In comparison, myotubes cultured in a well that also contains cancer cells seeded on a Transwell insert is shown schematically and photographically (Figures 1B,C). In each case, the medium was changed every 24 h. Medium was sampled for measuring secreted LIF at intervals described in section Materials and Methods.

A mouse specific ELISA was used to measure LIF levels in myotube cultures containing added cancer cell CM and in myotube cultures incubated with Transwell® inserts containing cancer cells. LIF levels in myotube cultures treated with one third volume of C26 CM had concentrations of 530 pg/ml at the beginning of a 24 h period of treatment and 370 pg/ml at the end of the 24 h period, immediately before the medium was changed (Figure 1D). Similar values were found at the beginning and end of the 48 and 72 h treatment periods in CM-treated myotube cultures (Figure 1D). The loss of LIF over the three 24 h periods was 30, 36, and 45% with the decrease likely due to the increasing area of unfused C2C12 cells growing in the wells, which was able to deplete more LIF during each incubation.

By comparison, in myotube cultures containing Transwells, LIF levels were not detectable at 0 and 1 h after incubation with Transwell® inserts containing C26 cells (Figure 1E). However, after 2 h of incubation, LIF levels were 7 pg/ml and then increased linearly to 110 pg/ml at 24 h immediately before the medium change (Figure 1E). The same pattern of LIF level was seen during the 24–48 h treatment period with the LIF levels increasing linearly from undetectable at 0 h to 195 pg/ml by 48 h. Finally, in the third 24 h period, the LIF was undetectable after the medium change and went up linearly to reach 290 pg/ml. As observed, the final levels were higher each day, likely because the number of tumor cells was increasing in the Transwell inserts. Thus, myotubes incubated with C26 CM were exposed to higher levels of LIF for a longer time compared to myotubes incubated with Transwell inserts containing C26 cancer cells. In the case of the one-time C26 CM addition, the average levels of LIF were 450, 410, and 370 pg/ml for the three 24 h periods. In the case of the C26 Transwells, the levels of LIF halfway through each 24 h period were 45, 125, and 180 pg/ml.

### The extent and progression of myotube atrophy treated with conditioned medium vs. transwell® inserts containing cancer cells

**Figure 2:**
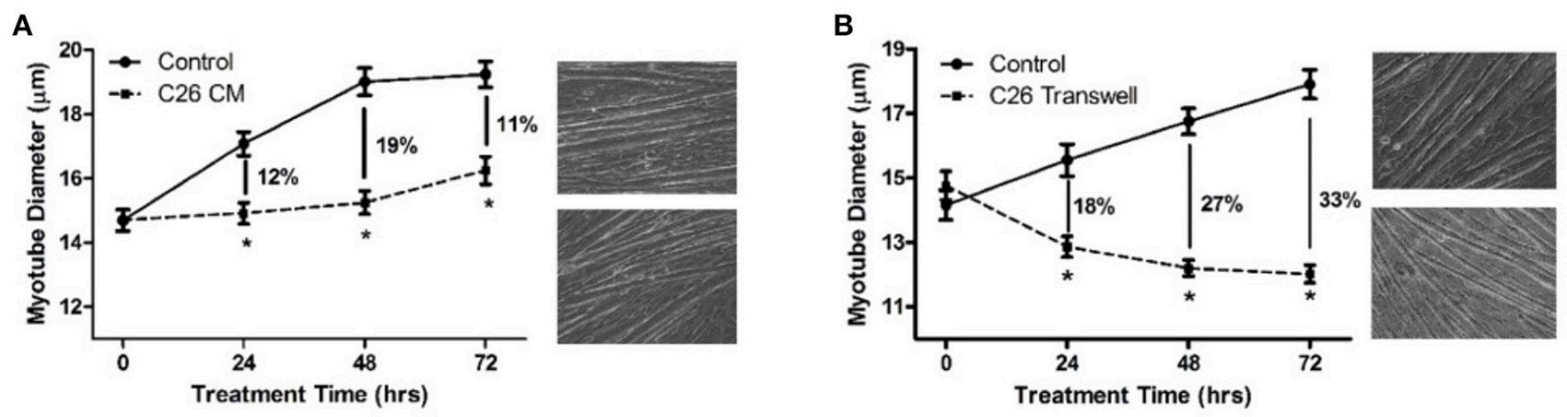
Myotube diameters in response to treatment with C26 CM or C26-Transwell inserts. **(A)** Treated with DM (control) or C26 CM for 72 h. DM or C26 CM was refreshed every 24 h and photographs of the wells were taken just before feeding. Percent atrophy indicated for each treatment time point in graph. **(B)** Treated with DM or Transwell inserts containing C26 tumor cells, medium refreshed every 24 h, again, photographs of the bottom wells taken just before feeding. On the right of each graph are photographs of phase images of representative wells of control (top) and treated (bottom) myotubes at 72 h. ^*^P < 0.05 compared to control value.

In order to determine the magnitude and progression of atrophy when myotubes are exposed to LIF in the two cases, myotube diameters were measured at 0, 24, 48, and 72 h of treatment by either the bolus CM or the Transwell® methods. Myotube atrophy was 12, 19, and 11% (*P* < 0.05) due to treatment with C26 CM at 24, 48, and 72 h, respectively, compared to control myotubes incubated in DM only (Figure 2A). Myotube atrophy was 18, 27, and 33% (*P* < 0.05) due to incubation with Transwell® inserts containing C26 cells at 24, 48, and 72 h, respectively, compared to control myotubes incubated with Transwell® inserts containing DM only with no cells (Figure 2B).

**Figure 3:**
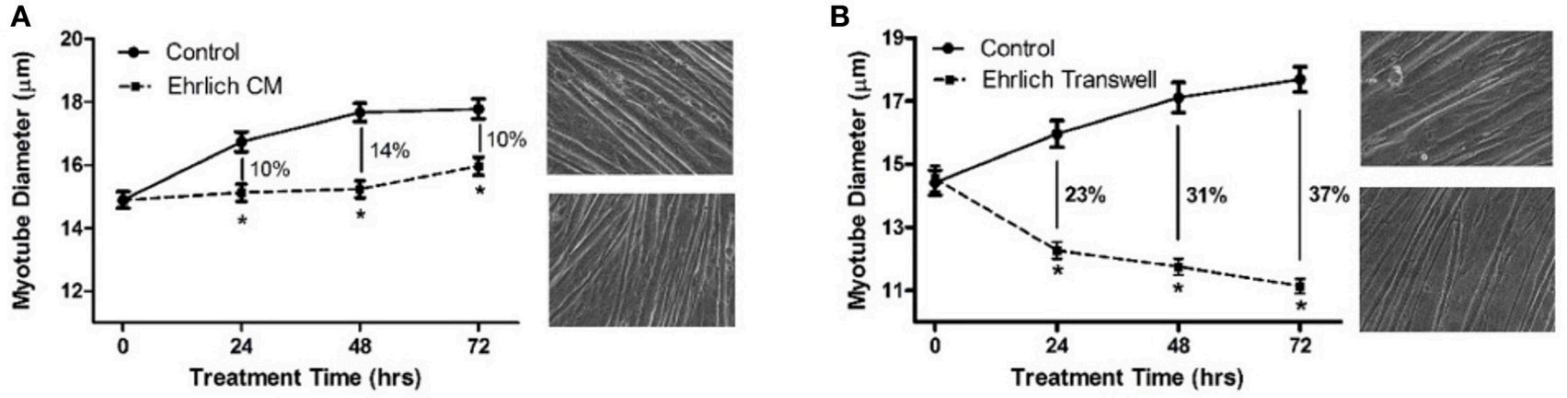
Myotube diameters in response to treatment with Ehrlich tumor cell CM or Ehrlich-Transwell inserts. **(A)** Treated with DM (control) or Ehrlich CM for 72 h. DM or Ehrlich CM was refreshed every 24 h, and photographs of the wells were taken just before feeding. Percent atrophy indicated for each treatment time point in graph. **(B)** Treated with DM or Transwell inserts containing Ehrlich tumor cells, medium refreshed every 24 h, again, photographs of the bottom wells taken just before feeding. To the right of each graph are photographs of phase images of representative wells of control (top) and treated (bottom) myotubes at 72 h. ^*^P < 0.05 compared to control value.

In order to test myotube atrophy from a second mouse tumor that is known for making high levels of LIF (Tomida et al., 1984), Ehrlich ascites carcinoma cells were used in the same two treatment designs as C26 to treat C2C12 myotubes. When exposed to CM made from Ehrlich ascites carcinoma cells, myotube atrophy was 10, 14, and 10% at 24, 48, and 72 h, respectively (Figure 3A). In comparison, when incubated with Transwell® inserts containing Ehrlich cells, myotube atrophy was 23, 31, and 37% at 24, 48, and 72 h, respectively (Figure 3B). Therefore, in both mouse carcinoma cell lines, there was a greater magnitude of atrophy when myotubes were exposed to cancer cell containing Transwell® inserts compared to exposure to CM made from cancer cells. Further, atrophy was progressively greater at each time point when myotubes were incubated with cancer cell containing Transwell® inserts compared to incubation with CM.

## Discussion

Skeletal muscle cells are deleteriously affected by tumor secretions as is the case with skeletal muscle, a primary target tissue in cancer cachexia. In fact, muscle loss, more than fat loss or body weight loss is the strongest predictor of morbidity and mortality (Martin et al., 2013). Prior to the present study, the way to study cancer cell secretions on muscle *in vitro* was to treat muscle cells in culture with solutions containing tumor cell CM. In fact, medium from many tumor cells causes wasting of cultured muscle cells, showing that tumor secreted factors are sufficient to cause atrophy independent from the host (Tisdale, 2009; Fearon et al., 2012). However, treatment of cultured myotubes with these secretions in a bolus fashion differs from the kinetics of tumor secretions and target tissue effects *in vivo*. From the time that the tumor begins to grow, target tissues are in continuous contact with secretions and the amounts steadily increase as the tumors grow in size (Seto et al., 2015) and, by metastasis, in number. Perhaps because the bolus addition of cancer cell CM differs from delivery *in vivo*, the extent of atrophy is less in myotube culture than in whole muscle in tumor-bearing mice or in humans. In the present study, we incubated myotubes with cancer cells contained in a compartment of the well separated by a porous membrane and fluid space to more closely recapitulate cachectic conditions *in vivo*, where the tumor cells and muscle fibers share a circulation but are not in contact. We found that the magnitude and progression of atrophy in myotubes exposed to cancer cells incubated in Transwell cultures was similar to mouse cancer cachexia found *in vivo* (e.g., Bonetto et al., 2011; Cornwell et al., 2014) and differed from myotubes treated with a bolus of cancer cell CM.

We recently showed that the cytokine LIF is completely responsible for myotube atrophy in a cell culture model of cancer cachexia (Seto et al., 2015). Only a validated blocking antibody against mouse LIF was able to inhibit C26 CM-induced C2C12 myotube atrophy, and it was 100% effective. In the present study, we found that over the 72 h of myotube treatment with addition of cancer cell CM, the LIF levels for each of the 3 successive 24-h treatment periods were higher over the entire period than at any time when myotubes were incubated with cancer cells plated on the Transwell insert. In the latter case, LIF levels began near zero during each 24-h treatment period and were highest at the end of the period before media replenishment. An average value for the Transwell cultures would not compare to that for the bolus treated cultures, since the LIF levels start each cycle at 0 pg/ml, but LIF was never higher than 300 pg/ml and more than half the time was < 150 pg/ml. The levels of LIF for the CM-treated cultures decreased slightly over each 24-h treatment period, but were always >220 pg/ml. The rate of increase of LIF in the Transwell cultures was 5, 8, and 12 pg/ml/h respectively for each of the 24-h treatment periods. As a comparison to *in vivo*, the increase in serum LIF during the final 6 days in C26 tumor-bearing mice, during which almost all of the muscle atrophy was found, amounted to 4 pg/ml per day (data from Seto et al., 2015).

The extent of myotube atrophy in the two models supports the idea that a constantly refreshed and increasing LIF delivery is more important than the total LIF level in producing myotube atrophy. Myotube atrophy was higher at each time point for the Transwell treatment and continued to increase throughout the 72 h while the bolus produced its maximal atrophy at 48 h and was less pronounced at 72 h. We interpret this to mean that the slowly increasing and continuous LIF level in the Transwell-containing plates is more effective at maintaining the pathways for atrophy than a bolus addition of CM containing a high LIF level. It is also possible that the large and sustained amount of LIF in the bolus treatment is too much for normal LIF signaling and causes downregulation of LIF receptors, but this is yet to be directly tested. However, the lessening of atrophy during the last 24-h period in the bolus case appear to reflect less responsiveness by the myotubes to the CM stimulus, which would be predicted if receptors had been reduced. The fact that myotube atrophy continued to increase over the 72 h of treatment with Transwell inserts suggests this is a more physiological model of cancer cachexia.

There are no previous studies on the use of Transwells to study tumor cell-induced muscle atrophy. There is a case in which myoblasts have been incubated with fibroblasts in a Transwell system thereby studying factors working in paracrine signaling between the cells (e.g., Rao et al., 2013), to regulate myoblast differentiation.

The most direct interpretation of the results in this study is that tumor cells plated on Transwell inserts deliver LIF in a continuously refreshed low to high dose pattern that is more effective at stimulating and maintaining the muscle atrophy exerted by tumors in a paracrine way. On the other hand, since both tumor cells and skeletal muscle cells are in continuous contact via the shared medium, there is the possibility that there is a paracrine cross-talk between the cells. Factor/s from the muscle cells might influence the secretions from the tumor to make different factors affecting the muscle. However, we chose C26 for this study because we have already shown that LIF explains all the effect of this tumor on skeletal muscle myotube diameter in cell culture (Seto et al., 2015). Furthermore, the difference in myotube atrophy between bolus and Transwell models was also observed and data presented, using Ehrlich tumor cells. The Ehrlich mouse tumor cells were the first identified source of LIF in a cancer context (Tomida et al., 1984). In future studies, however, our Transwell system makes a paracrine cross-talk study possible, and may be applied for other cases of tumor cell-induced muscle wasting.

The results presented here show that continuous delivery of cachectic factors from tumor cells is the best way to produce profound effects on skeletal muscle cell atrophy in cell culture and this model should be considered in addition to CM experiments, to dissect the tumor released factors responsible for wasting.

## Author contributions

RJ wrote a draft of the manuscript, conceived of design and collected, analyzed, and interpreted data. SK helped conceive of design, wrote parts of the manuscript, analyzed and interpreted data. JF and DS collected myotube diameter data for the CM experiments, RY and BZ collected myotube diameter data for the TW experiments and collected culture medium samples for the CM experiments, ME and KE collected culture medium samples for the TW experiments and measured myotube diameters for 72-h CM experiments.

### Conflict of interest statement

The authors declare that the research was conducted in the absence of any commercial or financial relationships that could be construed as a potential conflict of interest.
