# Corrigendum: Continuous Release of Tumor-Derived Factors Improves the Modeling of Cachexia in Muscle Cell Culture

**DOI:** 10.3389/fphys.2019.00394

**Published:** 2019-04-09

**Authors:** Robert W. Jackman, Jess Floro, Rei Yoshimine, Brian Zitin, Maythita Eiampikul, Khalid El-Jack, Danielle N. Seto, Susan C. Kandarian

**Affiliations:** Department of Health Sciences, Boston University, Boston, MA, United States

**Keywords:** muscle atrophy, myotubes, LIF, muscle wasting, C2C12, cancer, cachexia, transwell

An author name was incorrectly spelled as “Kahlid El-Jack.” The correct spelling is “Khalid El-Jack.”

The authors apologize for this error and state that this does not change the scientific conclusions of the article in any way. The original article has been updated.

